# Clinical-Like Cryotherapy in Acute Knee Arthritis Protects Neuromuscular Junctions of Quadriceps and Reduces Joint Inflammation in Mice

**DOI:** 10.1155/2022/7442289

**Published:** 2022-01-22

**Authors:** Paula A. T. S. Castro, Dafiner H. Machanocker, Genoveva F. Luna, Germanna M. Barbosa, Jonathan E. Cunha, Thiago M. Cunha, Fernando Q. Cunha, Thiago L. Russo, Tania F. Salvini

**Affiliations:** ^1^Department of Physical Therapy, Federal University of São Carlos, São Carlos, SP, Brazil; ^2^Department of Pharmacology, University of São Paulo, Ribeirão Preto, SP, Brazil

## Abstract

Rheumatoid arthritis is an autoimmune and inflammatory disease that affects synovial joint tissues and skeletal muscle. Clinical-like cryotherapy benefits signs of joint inflammation in knee osteoarthritis after 60 days of anterior cruciate ligament transection surgery. However, it is unknown whether it also benefits acute knee arthritis (e.g., reduces inflammatory process and protects neuromuscular junction [NMJ] and muscle fibers). We aimed to analyze the effects of clinical-like cryotherapy on NMJ and quadriceps muscle fibers in a model of acute knee arthritis. Twenty-four male C57BL/6 mice (20 to 25 g) were randomly allocated into three groups: control (mice with no intervention), antigen-induced arthritis (AIA; mice sensitized and immunized with intra-articular [i.a.] injection of methylated bovine serum albumin [mBSA]), and AIA+cryotherapy (mice sensitized, immunized with i.a. injection of mBSA, and submitted to a clinical-like cryotherapy protocol). Twenty-one days after sensitization, arthritis was induced in immunized mice via i.a. injection of mBSA (100 *μ*g/joint). Two clinical-like cryotherapy sessions (crushed ice pack for 20 min) were applied two hours apart. The first session was applied immediately after i.a. injection of mBSA. The quadriceps was removed two hours after the second clinical-like cryotherapy session for morphological analysis of muscle fibers (cross-sectional area), frequency distribution of muscle fiber area (%), and NMJ (area, perimeter, and maximum diameter). Gene expressions of mRNA involved in NMJ signaling (*γ*-nAChR, *α*1-nAChR, *ε*-nAChR, Agrin-MusK-Rapsyn, *α*-dystrobrevin, and utrophin) and atrophy (muscle RING-finger protein-1 and Atrogin-1) pathways were analyzed. Inflammatory signs were assessed in knee joint (swelling, articular surface temperature, and neutrophil migration in synovial fluid). Regarding morphological analysis of muscle fibers, 180 to 270 and >270 *μ*m^2^ classes were higher in the AIA+cryotherapy than the AIA group. Area, perimeter, and maximum diameter of NMJ also increased in the AIA+cryotherapy compared with the control group. Agrin mRNA expression increased in the AIA+cryotherapy compared with the control and AIA groups. In the atrophy pathway, Atrogin-1 increased compared with the control and AIA groups. The AIA+cryotherapy group reduced knee swelling and neutrophil migration compared with the AIA group. In conclusion, clinical-like cryotherapy increased Agrin expression, contributing to NMJ maintenance and increased Atrogin-1 expression, thus protecting NMJ and muscle fiber. Furthermore, clinical-like cryotherapy reduced inflammatory signs (swelling and neutrophil migration) of acute knee arthritis.

## 1. Introduction

Rheumatoid arthritis is a systemic autoimmune disease characterized by chronic inflammation of synovial tissues and production of nonorgan-specific antibodies, leading to bone and cartilage destruction [1, 2]. Other tissues may also be affected during disease progression, such as musculoskeletal, cardiovascular, and pulmonary [3, 4]. Although little is known about initial events or factors that perpetuate rheumatoid arthritis, advances in understanding the role of agents (i.e., cytokines, chemokines, growth factors, intracellular signaling molecules, and transcription factors) in its pathogenesis have contributed to development of new therapies [3, 5–7].

Ice is a nonpharmacological agent used to reduce pain and swelling during inflammatory response after injury [8]. Few studies in animal models of knee arthritis [9–13] and knee osteoarthritis (KOA) [14] assessed the effects of cryotherapy based on clinical practice. Since application of ice induces an inflammatory response, a clinical-like cryotherapy protocol based on clinical practice may contribute to maintaining and protecting the neuromuscular junction (NMJ) and morphology of quadriceps muscle fiber in acute knee arthritis.

A recent study by Cunha et al. [15] demonstrated atrophy of quadriceps (15.7%) and tibialis anterior (33%) muscles and increased Atrogin-1 and muscle RING-finger protein-1 (MuRF-1) in a KOA model (60 days after induction). Although the KOA group also showed NMJ remodeling (reduction of area and perimeter) in quadriceps and decreased NMJ diameter in tibialis anterior muscles [15], the acute effects of knee arthritis on morphology of quadriceps muscle and remodeling of NMJ are unknown. Moreover, there is a need to establish cryotherapy protocols based on clinical practice and understand the effects of joint inflammatory response on skeletal muscle. Therefore, the present study was aimed at analyzing the effects of clinical-like cryotherapy on NMJ and quadriceps muscle fibers in a model of acute knee arthritis.

## 2. Materials and Methods

### 2.1. Animals and Experimental Design

This study was performed using 24 male C57BL/6 mice (20 to 25 g), kept in temperature-controlled rooms (22 to 25°C), and provided with water and food *ad libitum*. Animal care and handling procedures followed the International Association for the Study of Pain guidelines and the Guide for the Care and Use of Laboratory Animals from the USA National Institute of Health. The study was approved by the research ethics committee of Ribeirão Preto Medical School from University of São Paulo (number 021/2016) and Federal University of São Carlos (number 1124010316/2015). Experimental analysis was divided into two periods: initial (immediately after intra-articular [i.a.] injection of methylated bovine serum albumin [mBSA]) and final (two hours after the second clinical-like cryotherapy session). Mice were randomly distributed into three groups (eight mice each): (1) control group: mice with no intervention; (2) AIA group: mice sensitized and immunized with i.a. injection of mBSA; and (3) AIA+cryotherapy group: mice sensitized, immunized with i.a. injection of mBSA, and submitted to the clinical-like cryotherapy protocol [16].

### 2.2. Antigen-Induced Arthritis

Mice were immunized as previously described [17–19]. AIA and AIA+cryotherapy groups were anesthetized (O_2_ 2 L/m and 2% isoflurane) one minute before each immunization to minimize pain. Then, mice were sensitized by subcutaneous injection on day zero with 500 *μ*g of mBSA in 0.2 mL of an emulsion containing 0.1 mL of saline and 0.1 mL of Freund's Complete Adjuvant (1 mg/mL of inactive *Mycobacterium tuberculosis*). Next, sensitization was boosted with the same preparation on days 7 and 14. Control mice were not immunized with mBSA. Twenty-one days after sensitization, arthritis was induced in immunized mice with i.a. injection of mBSA (100 *μ*g/joint) dissolved in 10 *μ*L of saline applied into the right tibiofemoral joint. Two hours after the second clinical-like cryotherapy session, all groups were anesthetized (O_2_ 2 L/m and 2% isoflurane) and euthanized by cervical dislocation.

### 2.3. Clinical-Like Cryotherapy Protocol

Mice received two sessions (20 min) of clinical-like cryotherapy, applied within two hours. The first session was applied immediately after i.a. injection. Mice were anesthetized (O_2_ 2 L/m and 2% isoflurane) for clinical-like cryotherapy and maintained in dorsal decubitus on a table with the right knee in the lateral position. Clinical-like cryotherapy consisted of applying a plastic pack filled with crushed ice (±5 g: 4.5 to 5.5 g) on the right knee (Figure S1). The ice pack was positioned on the central part of the knee (anterior, lateral, and medial portions) and replaced every 10 min each session; room temperature was controlled (±24°C) [16].

### 2.4. Morphological and Morphometric Analysis

#### 2.4.1. Muscle Fiber Cross-Sectional Area

Samples of quadriceps muscle were collected, quickly frozen in liquid nitrogen-cooled isopentane, and stored at -80°C before sectioning. Histological cryosections of the muscle (10 *μ*m) from all studied groups were cut and stained using the Toluidine Blue method. Cross-sectional area (CSA) was determined by measuring 100 muscle fibers (10 sequential fields at 20x magnification) from each mouse using a microscope attached to a computerized imaging analysis system (AxioVision 3.0.6 SP4, Carl Zeiss, Germany). All measurements were blindly assessed. CSA was also divided into classes (<70, 70 to 90, 90 to 180, 180 to 270, and >270 *μ*m^2^), according to Souza et al. [20]. Frequency distribution (%) of muscle fibers among classes corresponded to the ratio between number of fibers in each class and total number of fibers measured.

#### 2.4.2. NMJ Analysis Based on Nonspecific Esterase Technique

Longitudinal surface portions of the quadriceps muscle were trimmed to an end-plate motor portion (containing the motor point in proximal femoral region) and cut lengthwise in three or four slices. The resulting material was submitted to the nonspecific esterase technique [21] to characterize NMJ. Total area, perimeter, and maximum diameter were measured on 50 junctions of each mouse from all groups. Measures were analyzed by a blind researcher using the ImageJ software (version 1.53a, National Institutes of Health, USA).

#### 2.4.3. NMJ Analysis Based on Confocal Microscopy Technique

Mice were intracardially perfused with phosphate-buffered saline (PBS), followed by cold fixation (4% formaldehyde in PBS, freshly prepared) [20]. Quadriceps muscle was removed at femoral insertion and incubated with 0.1 M glycine for 30 min. Next, samples were washed and incubated with 1 *μ*g/mL rhodamine-*α*-bungarotoxin (Molecular Probes, Inc., USA) for 40 min at room temperature (24°C ± 1) to identify nicotinic acetylcholine receptors (nAChR). Muscles were washed, and each segment was analyzed using the laser-scanning confocal system (TCS SP5, Leica, Mannheim, Germany). Images were obtained with 20x objective lens (1.15 numeric aperture, ACS APO, Leica, USA), with constant pinhole to preserve thickness of the confocal plane. Intensity and gain were adjusted for each image using excitation wavelengths of argon-ion (488 nm) and krypton-ion (532 nm) lasers. Manual threshold of maximum intensity projection (pixel counting method) was performed in all digital images by adjusting the scale bar in the ImageJ software (version 1.53a, National Institutes of Health, USA). Approximately 30 end-plates of each muscle fiber type were measured to determine fiber proportion. Area, perimeter, and maximum diameter were compared between groups (Figure S2).

#### 2.4.4. Gene Expression of mRNA Involved in NMJ Signaling and Atrophy Pathways

Total RNA was extracted from samples of proximal quadriceps muscle and analyzed using TRIzol® Reagent (Life Technologies, USA), following manufacturer recommendations. RNA was quantified in the NanoVue™ Plus spectrophotometer (GE Healthcare, USA), and RNA purity was determined by measuring absorbance at 260 nm (RNA quantity) and 280 nm (protein quantity). Only samples with 260/280 nm (ratio > 1.8) were used. RNA integrity was evaluated using ethidium bromide staining (Invitrogen, USA), based on 28s and 18s ribosomal RNA. The extracted RNA was treated with DNase I Amplification Grade (Sigma Aldrich, AMPD, USA) to eliminate contamination with genomic DNA from samples. Next, mRNA reverse transcription was performed using iScript™ cDNA Synthesis Kit (Bio-Rad, USA), following manufacturer recommendations. mRNA expression levels were assessed through quantitative real-time polymerase chain reaction (PCR) using the CFX 96 Touch™ Real-Time PCR Detection System (version 3.0, Bio-Rad, USA). cDNA samples corresponding to mRNA of analyzed genes were amplified by the SsoFast™ EvaGreen® Supermix (Bio-Rad, USA), while primers were designed from sequences published in the GenBank (https://pubmed.ncbi.nlm.nih.gov/) using the Primer Express® 3.0.11 software (Applied Biosystems, USA) and synthesized by Life Technologies (USA) (Figure S3). Expression levels were normalized by glyceraldehyde 3-phosphate dehydrogenase, beta-2-microglobulin, beta cytoskeletal actin, and hypoxanthine-guanine phosphoribosyltransferase; expression was constant among all samples. Relative quantification of gene expression was performed using the comparative 2^∆∆^C(T) method [22].

#### 2.4.5. Joint Swelling

Tibiofemoral joint thickness (in mm) was measured two hours after the second clinical-like cryotherapy session and under anesthesia (O_2_ 2 L/m, 2% isoflurane) using an electronic digital caliper (Digimatic ABSOLUTE 150 mm, Mitutoyo, Japan). The mean of three measures was included in the data analysis [14, 15, 19].

#### 2.4.6. *In Vivo* Neutrophil Migration

The articular cavity of immunized (AIA and AIA+cryotherapy group) and nonimmunized (control group) mice was analyzed two hours after the second clinical-like cryotherapy session [16]. Leukocyte migration was assessed by washing articular cavities twice with 3.3 *μ*L of PBS containing 1 mM ethylenediaminetetraacetic acid (EDTA) and diluting to a final volume of 50 *μ*L with PBS/EDTA. Neutrophils were counted in a Neubauer chamber, diluted in Turk's solution. Cell counts were performed using a light microscope, and results were expressed as the number of neutrophils per joint cavity (mean ± standard deviation).

#### 2.4.7. Articular Surface Temperature

Thermographic images were obtained using a portable infrared camera (ThermaCAM® E320, 320 × 240-pixel resolution, 4x digital zoom, -0.10 to 25°C thermal sensitivity, ±2°C accuracy). Thermography was used to measure surface temperature (in °C) of the right quadriceps muscle two hours after the second clinical-like cryotherapy session. The reference point to analyze thermograph images of quadriceps muscle was 1 cm above and below and 0.5 cm lateral and medial to the patella. Laboratory temperature was maintained at 24°C for 12 hours before the procedure. Thermographic data were analyzed using the ThermaCAM® QuickReport software (version 1.1, USA). A blind researcher performed all measurements.

### 2.5. Statistical Analysis

Continuous variables were presented as mean ± standard deviation. Ordinary one-way ANOVA with Tukey's multiple comparison test compared intergroup differences. Repeated measure two-way ANOVA with Bonferroni post hoc test compared articular surface temperature and frequency distribution of quadriceps muscle fibers between groups. Statistical analyses were performed using GraphPad Prism, version 5.00 for Windows (GraphPad Software, California, USA). A *p* value of <0.05 was considered statistically significant.

## 3. Results

### 3.1. Morphological and Morphometric Analysis

#### 3.1.1. Muscle Fiber CSA

**Figure 1:**
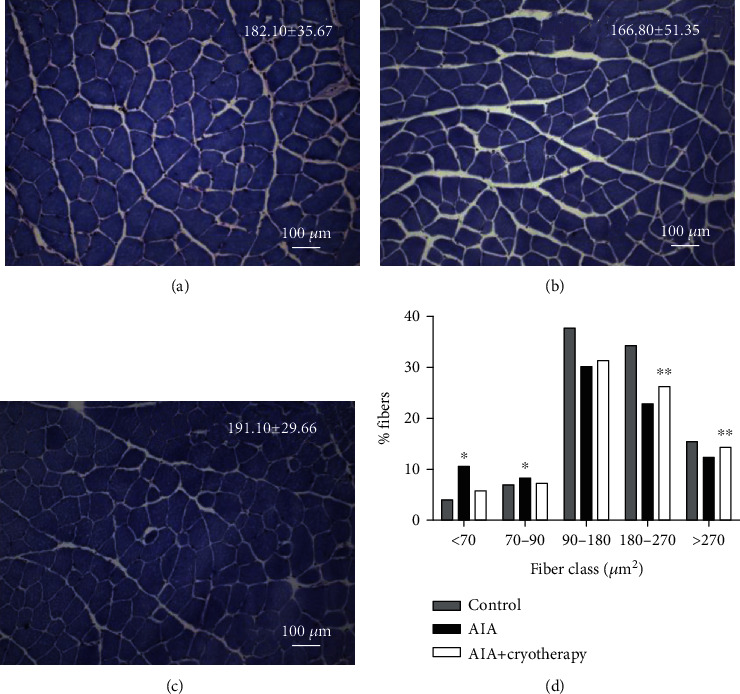
Cross-sectional area of muscle fibers in Toluidine Blue staining of mice from (a) control group, (b) AIA group, and (c) AIA+cryotherapy group. Scale bar: 100 *μ*m. Magnification: 20x. Values expressed as mean ± SD, *n* = 8, *p* = 0.46. (d) Frequency distribution of quadriceps muscle fibers. *p* < 0.001: ^∗^compared with control group; ^∗∗^compared with AIA group.

Morphology of the quadriceps muscle showed rounded polygonal fibers of different diameters, distributed in a mosaic pattern (Figure 1). No intergroup difference was observed between the CSA of muscle fibers (Figures 1(a)–1(c)). Frequency distribution of quadriceps muscle fibers was different only between classes (*p* < 0.001). AIA group increased frequency distribution in classes < 70 and 70 to 90 *μ*m^2^ and decreased in classes 90 to 180, 180 to 270, and >270 *μ*m^2^ compared with the control group (Figure 1(d)). An increase in classes 180 to 270 and >270 *μ*m^2^ was observed in the AIA+cryotherapy group compared with the AIA group (Figure 1(d)).

#### 3.1.2. NMJ Analysis Based on Nonspecific Esterase Technique

**Figure 2:**
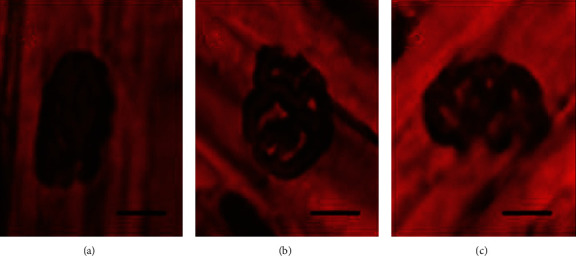
Representative image of NMJ morphology of quadriceps using nonspecific esterase technique: (a) control group; (b) AIA group; and (c) AIA+cryotherapy group. Scale bar: 10 *μ*m; magnification: 40x.

NMJ distribution in the quadriceps of all groups was morphologically characterized as a pretzel-like structure. Terminal branches of nerves were continuous, preserved, and without deformations or focal points in nAChR clusters. Also, the postsynaptic region demonstrated no prominent axonal sprouts (Figure 2). Area, perimeter, and maximum diameter were not different between groups (Table 1).

Values expressed as mean ± SD, *n* = 8. NMJ: neuromuscular junction; AIA: antigen-induced arthritis.

#### 3.1.3. NMJ Analysis Based on Confocal Microscopy Technique

**Figure 3:**
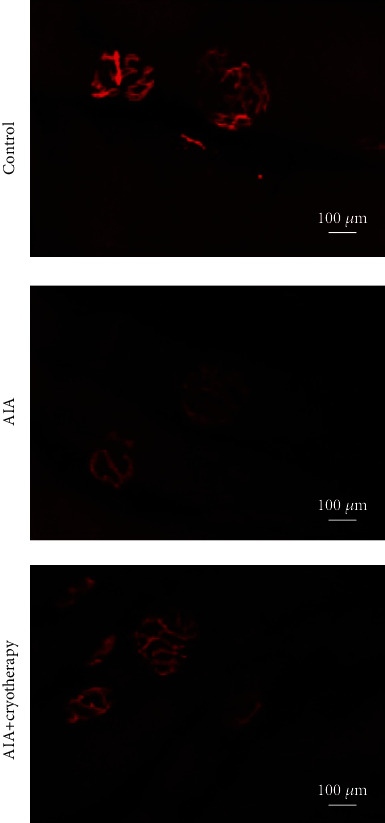
Representative image of NMJ morphology in quadriceps muscle using confocal microscopy technique. Panels illustrate postsynaptic nAChR (red) quantified in all groups. Scale bar: 100 *μ*m; magnification: 20x.

Confocal microscopy technique allowed the three-dimensional visualization of NMJ in the quadriceps muscle (Figure 3). NMJ showed a typical pretzel-like structure also observed in the nonspecific esterase technique. Area, perimeter, and maximum diameter of the quadriceps were higher in the AIA+cryotherapy group than the control group (Table 2). The perimeter was larger in the AIA group than in the control group (Table 2).

Values expressed as mean ± SD, *n* = 8. NMJ: neuromuscular junction; AIA: antigen-induced arthritis. ^∗^Compared with control group.

#### 3.1.4. Gene Expression of mRNA Involved in NMJ Signaling and Atrophy Pathways

Subunits *γ*-nAChR, *α*1-nAChR, and *ε*-nAChR were not different between groups. Among proteins (Agrin-MusK-Rapsyn) placed with nAChR, only Agrin showed increased expression compared with the control and AIA groups (Table 3). Regarding genes interacting with the intracellular domain of receptors and related to muscle atrophy (Atrogin-1 and MuRF-1), only Atrogin-1 presented increased expression compared with the control and AIA groups. For genes promoting nAChR stability in the synaptic region of the muscle (*α*-dystrobrevin and utrophin), none showed increased expression compared with the control group (Table 3).

Values expressed as mean ± SD, *n* = 8. AIA: antigen-induced arthritis. ^∗^Compared with control group, ^#^compared with AIA group.

#### 3.1.5. Joint Swelling

**Figure 4:**
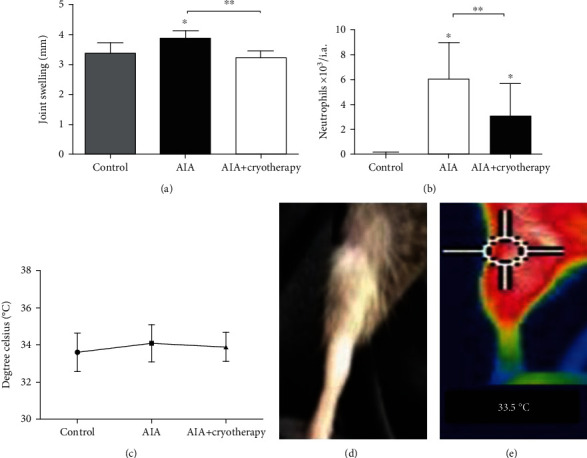
(a) Joint swelling (*p* < 0.001); (b) *in vivo* neutrophil migration (*p* < 0.001); (c) articular surface temperature (*p* = 0.59); (d) right paw of a mouse during temperature measurement; (e) region analyzed using thermography. Values expressed as mean ± SD, *n* = 8, ^∗^compared with Control group, ^∗∗^compared with AIA group.

Joint swelling decreased in the AIA+cryotherapy group compared with the AIA group two hours after the second clinical-like cryotherapy session (*p* < 0.001, Figure 4(a)). Joint swelling was also higher in the AIA group than the control group (*p* < 0.001, Figure 4(a)).

#### 3.1.6. *In Vivo* Neutrophil Migration

The AIA+cryotherapy group exhibited reduced neutrophil recruitment into knee joint (*p* < 0.001, Figure 4(b)) compared with the AIA group. In addition, the AIA group showed increased neutrophil recruitment into the knee joint two hours after the second clinical-like cryotherapy session compared with the control group (*p* < 0.001, Figure 4(b)).

### 3.2. Articular Surface Temperature

Articular surface temperature was not different between groups two hours after the second clinical-like cryotherapy session (Figure 4(c)).

## 4. Discussion

Clinical-like cryotherapy increased two proteins involved in the NMJ pathway and muscle atrophy: Agrin and Atrogin-1. Joint swelling and neutrophil migration into the synovial fluid were also reduced in mice with acute knee arthritis. The acute knee arthritis model of the present study showed no morphological changes in NMJ and quadriceps. A recent study from our group using a mouse model of KOA (eight weeks) damaged NMJ in quadriceps (reduced area, perimeter, and maximum diameter) and tibialis anterior muscles (reduced maximum diameter) [15]. The short period to induce joint inflammation possibly avoided impairment of terminal motor activity in acute knee arthritis. Moreover, this induction possibly contributed to absence of quadriceps muscle atrophy since no alteration in CSA was found, despite increased Atrogin-1 levels.

Although clinical-like cryotherapy did not alter synaptic NMJ receptors (*γ*-nAChR, *α*1-nAChR, and *ε*-nAChR subunits), it increased Agrin expression, a stability protein of nAChR in the synaptic region. Agrin maintains the receptor cluster in the postsynaptic region, binds to MusK-Rapsyn for stability and maintenance of NMJ [23], allows proper interactions between nerve terminal and muscle fibers, and delays the onset of muscle atrophy [20, 24]. In the present study, clinical-like cryotherapy after acute-induced knee joint inflammation increased Agrin expression, which possibly contributed to maintaining and protecting quadriceps NMJ and reducing inflammatory signs of synovial fluid (edema and neutrophil migration). A previous study conducted with a model of chronic inflammation (sepsis) showed that reduced Agrin expression contributed to abnormal expression of nAChR and caused neuromuscular dysfunction [25]. Therefore, the increase in Agrin expression is also related to activation of the immunoinflammatory pathway [26, 27], regulating the expression of IL-1*β* and TNF-*α* cytokines released at high levels in the AIA model.

An increase in Atrogin-1 in the AIA model was associated with weakness [28] and muscle atrophy [29]. According to Bodine et al. [30], muscle wasting in arthritis is associated with increased Atrogin-1 expression, as observed in denervation, immobilization, and suspension of hind limbs. In addition, an important catabolic effect of arthritis (i.e., increased muscle proteolysis) indicates increased expression of atrogenes [29]. This catabolic effect and time (i.e., few hours after induction of knee joint inflammation) were possibly not enough to promote morphological changes in quadriceps muscle fibers. Another 8-week arthritis model increased Atrogin-1 and quadriceps muscle atrophy [15], indicating that time after KOA induction was important for atrophy.

Atrophy occurs through a set of adaptations and increases proteolysis [31]. Identification of signaling pathways that regulate Atrogin-1 expression, such as insulin-like growth factor-1 (IGF-1) [29] and myogenic transcription factors [29], may explain atrophy in acute knee arthritis. Other NF-*κ*B-independent signaling pathways and FoxO1-dependent mechanisms [32] need to be investigated using this experimental model.

Stable microtubules regulate focal insertion of nAChR in the postsynaptic membrane and may be a protective factor for NMJ [33]. The function of microtubules is related to maintenance and stability of NMJ formation; however, literature describing their function is still scarce [33]. Furthermore, the expression of different IGF-1 isoforms (e.g., IGF-1Ea and IGF-1Eb) may be another protective factor against degeneration of NMJ, as observed in age-related experiments [34]. However, we did not fragment NMJ, and further studies using AIA are needed to elucidate this question. Thus, clinical-like cryotherapy increased the expression of both Atrogin-1 and Agrin, promoting immunoinflammatory and neuromuscular signaling and protection of NMJ and quadriceps muscle fibers.

Clinical-like cryotherapy reduced signs of joint inflammation in a mouse model of KOA, decreasing the number of leukocytes and levels of cytokines in the synovial fluid [14]. In a previous study by Castro et al. [16], clinical-like cryotherapy in a mouse model of acute knee arthritis also showed decreased inflammatory signs of joint swelling, neutrophil migration, and levels of cytokines IL-6, IL-1*β*, and TNF-*α* in the synovial fluid [16]. These results [16] indicated that cryotherapy (widely used in clinical practice with patients with osteoarthritis) [35] might be a nonpharmacological procedure to control inflammation in acute arthritis.

## 5. Conclusions

Clinical-like cryotherapy in acute knee joint increased the expression of Atrogin-1 and Agrin, contributing to maintaining NMJ of quadriceps and reducing inflammatory signs of synovial fluid (i.e., swelling and neutrophil migration).

## 6. Study Limitations

This study had some limitations. Neither sham nor placebo groups were included in experimental analyses. Moreover, the control group did not use anesthesia or experience stress as the AIA and AIA+cryotherapy groups. The studied period was short (acute) to analyze NMJ and quadriceps muscle fiber alterations.

## Figures and Tables

**Table 1:** Area, perimeter, and maximum diameter of quadriceps after morphometric analysis of NMJ using nonspecific esterase technique.

	Control	AIA	AIA+cryotherapy	*p* value
NMJ total area (*μ*m^2^)	58.91 ± 13.02	65.61 ± 11.30	63.89 ± 9.47	0.50
NMJ perimeter (*μ*m)	31.23 ± 3.55	32.84 ± 1.95	32.15 ± 2.63	0.53
NMJ maximum diameter (*μ*m)	11.13 ± 1.27	11.41 ± 1.09	11.38 ± 1.02	0.87

**Table 2:** Area, perimeter, and maximum diameter of quadriceps after morphometric analysis of NMJ using confocal microscopy technique.

	Control	AIA	AIA+cryotherapy	*p* value
NMJ total area (*μ*m^2^)	307.80 ± 92.80	380.40 ± 214.6	446.50 ± 252.00^∗^	0.03
NMJ perimeter (*μ*m)	131.60 ± 39.18	198.10 ± 101.90^∗^	219.3 ± 115.00^∗^	<0.001
NMJ maximum diameter (*μ*m)	28.07 ± 6.34	32.93 ± 11.29	34.73 ± 12.92^∗^	0.04

**Table 3:** Gene expression of mRNA involved in NMJ signaling and atrophy pathways.

	Control	AIA	AIA+cryotherapy	*p* value
	(*n* = 8)	(*n* = 8)	(*n* = 8)	
*γ*-nAChR	1.00 ± 0.75	1.23 ± 0.44	1.29 ± 0.68	0.63
*α*1-nAChR	1.00 ± 0.28	1.13 ± 0.65	1.39 ± 0.44	0.29
*ε*-nAChR	1.00 ± 0.59	1.52 ± 0.95	2.02 ± 2.22	0.37
Agrin	1.00 ± 0.35	1.88 ± 0.92	3.72 ± 1.92^∗^^#^	<0.001
MusK	1.00 ± 0.39	0.95 ± 0.26	1.43 ± 0.63	0.09
Rapsyn	1.00 ± 0.36	1.96 ± 1.08	2.36 ± 1.68	0.08
MuRF-1	1.00 ± 0.71	1.18 ± 0.53	1.93 ± 1.03	0.06
Atrogin-1	1.00 ± 0.34	2.37 ± 0.75	5.59 ± 1.77^∗^^#^	<0.001
*α*-Dystrobrevin	1.00 ± 0.83	0.77 ± 0.89	1.34 ± 0.60	0.36
Utrophin	1.00 ± 0.52	1.52 ± 0.75	1.87 ± 0.75	0.05

## Data Availability

The research data used to support the findings of this study are available from the corresponding author upon request.

## Supplementary Materials

Supplementary Materials Figure S1: mouse position during clinical-like cryotherapy session. Position of the mouse and ice pack during clinical-like cryotherapy session and anesthesia with isoflurane. Figure S2: user guide: using confocal microscopy for NMJ analysis. This user guide comprises a workflow designed to be used with Figure 3 and Table 2. The following descriptions guide the user through analysis of each NMJ using ImageJ software. Figure S3: oligonucleotide primers used for real-time PCR amplification of reverse-transcribed RNA. Table with all oligonucleotide primers used in this study, including nicotinic acetylcholine receptors, neuromuscular signaling, atrophy pathways, and reference genes.
