# Supplementary material for: Clinical-Like Cryotherapy in Acute Knee Arthritis Protects Neuromuscular Junctions of Quadriceps and Reduces Joint Inflammation in Mice
Source: Biomed Res Int. 2022 Jan 22;2022:7442289. doi: 10.1155/2022/7442289 (PMC8800614; doi:10.1155/2022/7442289)
Supplement: Supplementary Materials — Figure S1: mouse position during clinical-like cryotherapy session. Position of the mouse and ice pack during clinical-like cryotherapy session and anesthesia with isoflurane. Figure S2: user guide: using confocal microscopy for NMJ analysis. This user guide comprises a workflow designed to be used with Figure 3 and Table 2. The following descriptions guide the user through analysis of each NMJ using ImageJ software. Figure S3: oligonucleotide primers used for real-time PCR amplification of reverse-transcribed RNA. Table with all oligonucleotide primers used in this study, including nicotinic acetylcholine receptors, neuromuscular signaling, atrophy pathways, and reference genes. [file 7442289.f1.pdf]

Supplementary Materials

Figure S1 - Mouse positioned during the cryotherapy session

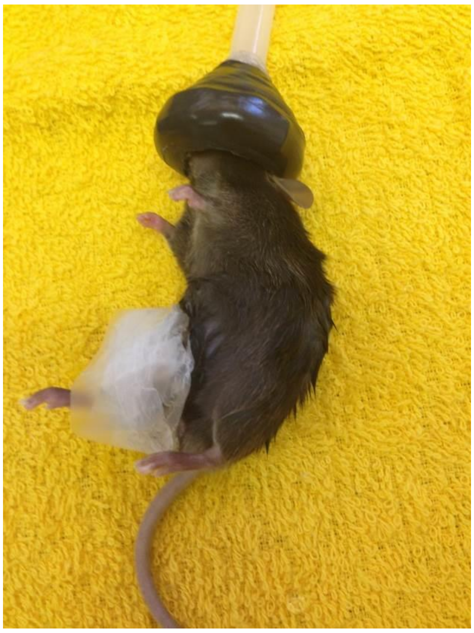

Figure S2 - User's Guide: Using confocal microscopy for analysis of neuromuscular junction (NMJ)

This user guide comprises a workflow designed for use in conjunction with Figure 3 and Table 2 of the manuscript. The following descriptions guide the user through the analysis steps of each NMJ using *Image J software*. The instructions in italics refer to the functions selectable from the *Image J software* menus. See the manuscript for further explanations and definitions of individual terms. Contact Castro, P.A.T.S. by email: paula.soupat@gmail.com.

1.Determining the working scale

Open your image by *File - Open*, then on the bar scale of your figure make an overlap of the size using the / *(straight line)* icon. Click *Analyze - set scale* and enter the bar scale value in the *Know distance* item and set the *unit of length in µm* item. To analyze all the figures in the study, click *Global* and standardize the scale of the complete experiment. Click on *Analyze - set measurements* and define which statistical measures you want to use to analyze your study of the figure (in our study, *area* parameters were used for Figure 3).

2.Selecting your image

After viewing the RGB image, via *Image - Color - Split channel,* select the channel (*red, green or blue)* you want to work on.

3.Treshold Targeting

Particle analysis requires the image to be binary. For this image transformation, *Image - Adjust - Threshold*. Pixels within the threshold range are displayed in red. The user changes to *Dark background - Apply* and then the image threshold is converted to a binary image.

4. Defining the background area

To define the *area,* draw a rectangle in the *background area* and press *Ctrl+C*, then go to *File - New - Internal Clipboard*. A *Clipboard* window will open. In the *Clipboard* window, press *Ctrl+M* and save *the result of the background area* (it will be used as a normalizer).

5. Definition of total sample area

Analysis of the *sample area* requires the parameters of the *Analyze - Set scale* in *area*. To define the area, draw a rectangle in the desired *sample area* and press *Ctrl+C*, then go to *File*

- *New* - *Internal Clipboard*. A Clipboard window will open. In the *Clipboard window*, press *Ctrl+M* and save the result of the *sample area*.

6.Definition of fragmented area

With the same rectangle established for the *sample area*, the *fragmented area* is defined using the *Wand (tracing) tool*. In the *Clipboard window,* press *Ctrl+M* and save the result of the *fragmented area*.

Figure S3 - Oligonucleotide primers used for real-time PCR amplification of reverse transcribed RNA.

**Housekeeping**	GAPDH foward	CCTTTTCAAGCGTCTTCCTG
	GAPDH reverse	CTCCTGGCTTCTGTCTTTGG
	HPRT foward	GTGCAACCATTGCCCTAAGT
	HPRT reverse	CAGCCAGCATCTCAGGTGTA
	BETA ACTINA foward	GACGGCCAGGTCATCACTAT
	BETA ACTINA reverse	AAGGAAGGCTGGAAAAGAGC
	B2M foward	ATGGGAAGCCGAACATACTG
	B2M reverse	CAGTCTCAGTGGGGGTGAAT
**Nicotinic Acetylcholine Receptors (nAChR)**	γ-nAChR foward	CCACCAGAAGGTGGTGTTCT
	γ-nAChR reverse	TGAGGAGATGAGCACACAGG
	α1-nAChR foward	TCCCAGCATCTAGCACACAG
	α1-nAChR reverse	GAAAGGCACCACCAAACAGT
	ε-nAChR foward	CCAGGAAACCCTCTTCTTCC
	ε-nAChR reverse	ATGACTGTGGAAGGGTCAGG
**Neuromuscular Signaling Pathway**	AGRIN foward	GCACACCTTCGAGAGAGACC
	AGRIN reverse	CATGTGGAGTTGTGGGAGTG
	MUSK foward	AGCCGATGTGTCTGCTCTTT
	MUSK reverse	AGTGTGCGGGGACATACTTC
	RAPSYN foward	ACGGACAGGTCCACAAACTC
	RAPSYN reverse	AGCAGGTCCAGCCTCTACAA
**Atrophy Pathway**	MURF-1 foward	CTCCCCTTTGTGGTGTGTCT
	MURF-1 reverse	GAGGCAGGAGGCACACTTAG
	ATROGIN-1 foward	CTCACGGAACACTTTGCTGA
	ATROGIN-1 reverse	CACCTTCACCTGACACATGG
	α-DISTROBREVIN foward	CCTATCTGCACCAGCCTAGC
	α-DISTROBREVIN reverse	AGCGGAGTTAGGCACTGAAA
	UTROPHIN foward	AGCACAGGGAAGCTGGAGT
	UTROPHIN reverse	GGAAGTTGAAGCAGGTGAGC
